# Tuberous sclerosis complex neuropathology requires glutamate-cysteine ligase

**DOI:** 10.1186/s40478-015-0225-z

**Published:** 2015-07-30

**Authors:** Anna R. Malik, Ewa Liszewska, Agnieszka Skalecka, Malgorzata Urbanska, Anand M. Iyer, Lukasz J. Swiech, Malgorzata Perycz, Kamil Parobczak, Patrycja Pietruszka, Malgorzata M. Zarebska, Matylda Macias, Katarzyna Kotulska, Julita Borkowska, Wieslawa Grajkowska, Magdalena E. Tyburczy, Sergiusz Jozwiak, David J. Kwiatkowski, Eleonora Aronica, Jacek Jaworski

**Affiliations:** Laboratory of Molecular and Cellular Neurobiology, International Institute of Molecular and Cell Biology, Warsaw, 02-109 Poland; Department of (Neuro)Pathology, Academic Medical Center, Amsterdam, 1105 AZ The Netherlands; Swammerdam Institute for Life Sciences and Center for Neuroscience, University of Amsterdam, Amsterdam, 1090 GE The Netherlands; Stichting Epilepsie Instellingen Nederland (SEIN), Heemstede, 2103 SW The Netherlands; Department of Neurology and Epileptology, Children’s Memorial Health Institute, Warsaw, 04-730 Poland; Division of Pulmonary Medicine and of Genetics, Brigham and Women’s Hospital, Harvard Medical School, Boston, MA 02-115 USA; Department of Child Neurology, Medical University of Warsaw, 02-091 Warsaw, Poland

**Keywords:** Tuberous Sclerosis Complex, Glutamate-cysteine ligase, Cellular stress, Brain tumors, Cell death

## Abstract

**Introduction:**

Tuberous sclerosis complex (TSC) is a genetic disease resulting from mutation in *TSC1* or *TSC2* and subsequent hyperactivation of mammalian Target of Rapamycin (mTOR). Common TSC features include brain lesions, such as cortical tubers and subependymal giant cell astrocytomas (SEGAs). However, the current treatment with mTOR inhibitors has critical limitations. We aimed to identify new targets for TSC pharmacotherapy.

**Results:**

The results of our shRNA screen point to glutamate-cysteine ligase catalytic subunit (GCLC), a key enzyme in glutathione synthesis, as a contributor to TSC-related phenotype. GCLC inhibition increased cellular stress and reduced mTOR hyperactivity in TSC2-depleted neurons and SEGA-derived cells. Moreover, patients’ brain tubers showed elevated GCLC and stress markers expression. Finally, GCLC inhibition led to growth arrest and death of SEGA-derived cells.

**Conclusions:**

We describe GCLC as a part of redox adaptation in TSC, needed for overgrowth and survival of mutant cells, and provide a potential novel target for SEGA treatment.

**Electronic supplementary material:**

The online version of this article (doi:10.1186/s40478-015-0225-z) contains supplementary material, which is available to authorized users.

## Introduction

Tuberous sclerosis complex (TSC) is a common neurocutaneous disease, inherited in an autosomal dominant pattern, affecting 1:6,000 live births [29]. It is caused by mutations in either of two genes encoding components of the TSC protein complex: TSC1 (hamartin) and TSC2 (tuberin). Disrupted TSC complex function results in overactivation of mammalian Target of Rapamycin complex 1 (mTORC1), an important regulator of cell metabolism [26]. mTORC1 overactivation is believed to be the main cause of the pathological overgrowth of cells and development of tubers in different tissues, including the brain. Patients develop 3 types of brain lesions: subependymal nodules, cortical tubers and subependymal giant cell astrocytomas (SEGAs) of glio-neuronal origin [4, 27]. The last two contribute to TSC clinical symptoms—cortical tubers are considered as epileptogenic [18], while growth of SEGA is commonly associated with the development of hydrocephalus and can lead to severe complications, including death [30]. Extensive research concerning tuberous sclerosis complex has led to a breakthrough in the treatment of this disease, enabling pharmacotherapy with mTOR inhibitors [20, 30]. mTOR inhibitors reportedly decrease SEGA size, and everolimus was recently approved by the FDA and EMA for TSC-associated SEGAs [34]. Moreover, many reports indicate that mTOR inhibition may alleviate epilepsy severity in TSC patients [21, 44]. However, withdrawal of mTOR inhibitor is associated with rapid tumor regrowth, and mTOR inhibitors use is burdened with significant adverse events, including infections, mouth ulcers, and hyperlipidemia [38]. In some cases, use of mTOR inhibitors can even lead to life-threatening complications, including sepsis and death [41]. Thus, the development of a more efficient and safer drug is of utmost importance.

Therefore, in this study, we aimed to provide new potential drug targets in TSC pharmacotherapy by identifying proteins crucial for the pathological overgrowth typical of TSC. We report results of an shRNA screen, in which we silenced expression of genes potentially linked to TSC pathology or mTOR signaling. We identified several proteins that possibly contribute to TSC-related phenotype. Among them, we focus on the role of glutamate-cysteine ligase catalytic subunit (GCLC), a protein important for the control of oxidative stress levels. We show that GCLC inhibition increases cellular stress and reduces mTOR hyperactivity in TSC2-depleted neurons and SEGA-derived cells. Moreover, we provide evidence that this protein might be a potential target in the pharmacotherapy of TSC.

## Materials and methods

### Drugs and antibodies

All drugs and antibodies used for the study are described in Supplementary materials and methods (Additional file 1).

### DNA constructs

pCx EGFP-N1, pSuper and β-actin-GFP [19] plasmids have been described previously [2, 3, 19]. The sequences encoding shRNAs used in the screen were cloned into the pSuper vector and are listed in Additional file 2: Table S1. mRNA sequences targeted in TSC2 and GCLC as well as cloning strategy for TSC2 and its shRNA-resistant mutants is described in Supplementary materials and methods (Additional file 1).

### Neuronal cultures, transfection, drug treatments, and immunofluorescence

The animals used to obtain neurons for tissue cultures were sacrificed according to the protocols approved by the First Ethical Committee in Warsaw, Poland (permission #75/2010), which are in compliance with the European Community Council Directive (86/609/EEC). Primary cortical cultures were prepared from embryonic day 19 (E19) rat brains as described previously [25]. For the purpose of immunofluorescence and morphometric analysis, neurons were transfected on Day 6 *in vitro* (DIV6) with Lipofectamine2000 as described earlier [35]. Twenty nanomolar rapamycin or 10 μM L-BSO were added to the medium 2 h after transfection and the medium was not changed until the end of the experiment. Neurons were fixed with 4 % paraformaldehyde (PFA) and 4 % sucrose in PBS at DIV10. Immunofluorescent staining was performed as described recently [25].

When a highly efficient gene transfer was required (Western blot), plasmids were introduced to freshly isolated neurons (DIV0) using the AMAXA nucleofection procedure (Lonza, Basel, Switzerland) as described recently [25]. For GCLC inhibition in nucleofected cells, 10 μM L-BSO was added to the medium 18 h post nucleofection. Neurons were lysed 30 h later.

### SEGA-derived cell culture and treatment

The study was approved by The Ethics Board at the Children’s Memorial Health Institute, Warsaw, Poland. The samples of patients’ SEGAs were analyzed after written consent was obtained from their parents. Patients were diagnosed as having TSC according to Roach’s criteria. The patients presented with acute hydrocephalus and were operated after large SEGAs were revealed in brain MRI (Additional file 3: Figure S3a). Freshly resected SEGA samples from two patients were cut into small pieces and trypsynized for 1 h at 37 °C. After trypsinization the tissue fragments were dispersed with a pipette to small clumps or single cells. The obtained cell suspension was centrifuged and the pellet was suspended in DMEM 4.5 g/l glucose supplemented with 5 % fetal bovine serum (FBS; Gibco, Karlsruhe, Germany) and antibiotics (100 U/ml penicillin, 100 μg/ml streptomycin; Sigma, St. Louis, MO). Cells were maintained for about 2 weeks until they reached confluence and were used for experiments.

For live imaging experiments, cells were plated on gelatin-coated μ-Slide VI 0.4 plates (Ibidi, Planegg, Germany). During pharmacological treatment, the medium was changed every second day and the drugs were used as follows: U0126 (20 μM), rapamycin (20 nM), L-BSO (20 or 100 μM).

### COS-7 cell culture and transfection

COS-7 cells (ATCC) were cultured in DMEM supplemented with 10 % FBS and antibiotics. Cells were transfected using Lipofectamine2000 (Invitrogen, Carlsbad, CA) according to manufacturer protocol.

### shRNA library screen

Target genes covered by the shRNA library are listed in Supplementary materials and methods (Additional file 1). The majority of targets was selected based on published data [5, 6, 45]; for more detailed description see Supplementary materials and methods (Additional file 1). Whenever possible, 3 shRNAs were designed against given mRNA and shRNAs coding sequences and cloned into the pSuper vector. However, in some cases, only two shRNAs could be designed.

In screening experiments, cortical neurons were transfected on Day 6 *in vitro* (DIV6) with TSC2sh together with pools of pSuper plasmids that encoded shRNAs targeting a given gene and β-actin-GFP. Each culture plate contained 3 control variants: (i) transfected with pSuper/β-actin-GFP, (ii) transfected with TSC2sh/pSuper/β-actin-GFP, and (iii) transfected with TSC2sh/pSuper/β-actin-GFP and treated with 20 nM rapamycin. Four days after transfection, the neurons were fixed and cell images were acquired. To avoid variability caused by differences between cultures, the area of neuron soma was quantified as a percentage of the mean value obtained for neurons in pSuper/β-actin-GFP control variant from the same experimental plate. Two independent screening experiments were performed, and the mean value was calculated from both of them for each shRNA pool.

### In vivo electroporation in neonates

All the procedures were approved by the First Ethical Committee in Warsaw, Poland (permission #569/2014), which is in compliance with the European Community Council Directive (86/609/EEC). Neonates (P0; Wistar, both sexes) were anesthetized by hypothermia and the plasmid solution was injected to the right lateral ventricle. Next, the animals were subjected to electrical pulses. Electroporated animals were warmed on a heating pad for several minutes before being returned to the mother. For detailed electroporation protocol, rat tissue preparation and staining procedures see Supplementary materials and methods (Additional file 1).

### Human tissue samples

The cases included in this study were obtained from the archives of the departments of neuropathology of the Academic Medical Center (University of Amsterdam) and the University Medical Center in Utrecht (UMCU). We examined 10 surgical specimens, 5 cortical tubers (male/female: 3/2; mean age at surgery: 16.8 years, range: 10–23), and 5 subependymal giant-cell astrocytomas (SEGA; male/female: 3/2; mean age at surgery: 12 years, range: 1–23) from patients undergoing epilepsy surgery or surgery for obstructive hydrocephalus. Informed consent was obtained for the use of brain tissue and for access to medical records for research purposes. Tissue was obtained and used in a manner compliant with the Declaration of Helsinki. For the SEGA, we used the WHO classification of tumors of the central nervous system [24]. All patients fulfilled the diagnostic criteria for TSC [11]. All patients included had TSC2 mutations. Normal-appearing control cortex was obtained at autopsy from 5 age-matched patients without history of seizures or other neurological diseases. Autopsied brain tissues from patients with Alzheimer’s disease (AD) and trauma (as well as skin specimens) were also examined (as positive controls for immunohistochemical analysis; AD and trauma specimens (for HO-1 and Hsp70) and skin specimens (for GCLC)). All autopsies were performed within 24 h after death. Histologically normal temporal neocortex (without evidence of significant neuronal loss, gliosis, or malformation) from two patients undergoing extensive surgical resection of the mesial structures for the treatment of medically intractable complex partial epilepsy was also used for immunocytochemical analysis*.* For human tissue preparation and staining procedures, see Supplementary materials and methods (Additional file 1).

### Image analysis and quantification

Olympus Cell^R station was used to obtain neuron images in screening experiments and for live imaging of SEGA-derived cells. Confocal images of immunofluorescently stained cortical neurons and rat brain sections were obtained with a Zeiss LSM710NLO microscope. The morphometric analysis of neuron soma area and immunofluorescence intensity analysis were performed via MetaMorph image analysis software (Universal Imaging Corporation, Downingtown, PA) with Integrated Morphometry Analysis and Region Measurements functions, respectively. For the analysis of SEGA-derived cells’ area, ImageJ software was used. For 3D reconstruction and morphometric analysis of GFP positive cells from rat brain slices, Imaris software (Bitplane, Zurich, Switzerland) was used.

### Statistical analysis

The data concerning neurons were obtained from three batches of cells, with the exception of the screening experiment that was performed on two batches of neurons. SEGA live imaging was performed twice for each of two independent SEGA-derived cultures. For animal studies, we reduced the number of animals to the minimum required for reliable statistics. Animals were randomly allocated to experimental groups and no blinding was done. The specimens were coded for microscopy analysis. The statistical analyses were performed using Prism Software (GraphPad, San Diego, CA) and Kruskal-Wallis test followed by Dunn’s *post hoc* test, or two-way ANOVA depending on the type of data analyzed. In the latter case variance was similar between compared groups. The number of values (analyzed cells) and animals in experimental groups is provided in Supplementary materials and methods (Additional file 1).

## Results

### Screening experiments reveal new proteins indispensable for abnormal growth of TSC2-depleted neurons

**Fig. 1:**
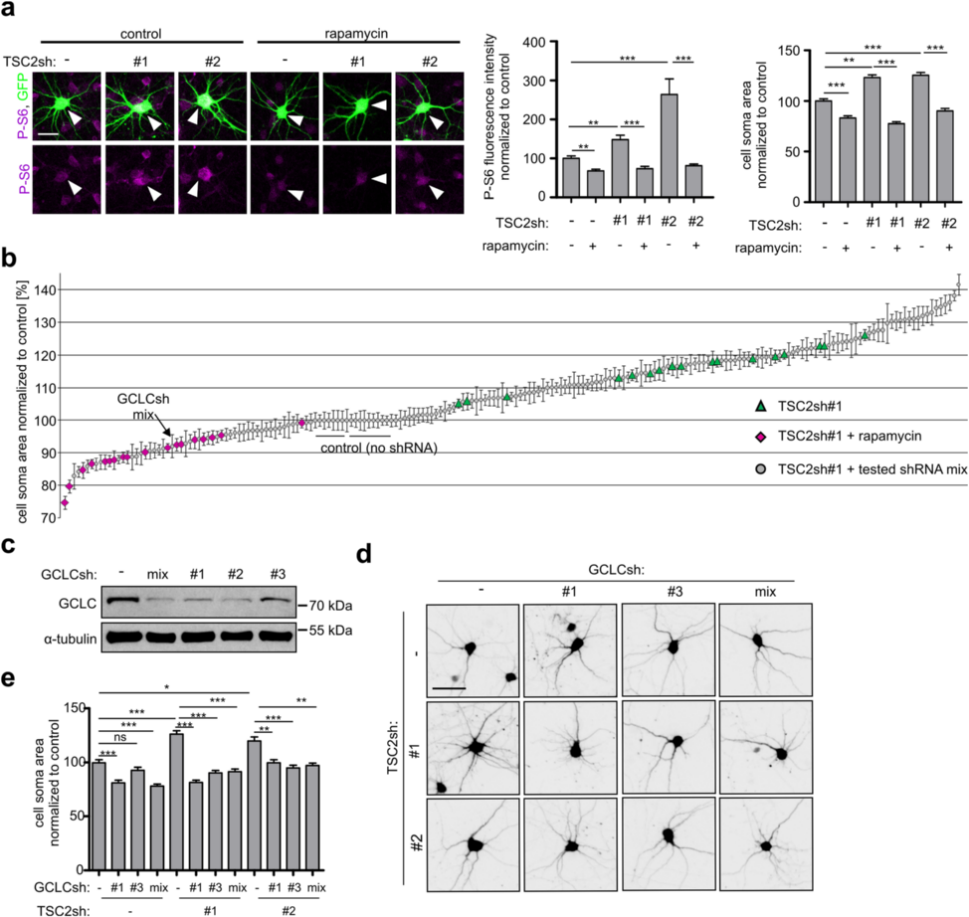
GCLC knockdown reverses the phenotype of model dysmorphic neurons. **a** TSC2 knockdown in rat cortical neurons as a model of TSC-related dysmorphic neurons. Neurons were transfected with empty pSuper vector, TSC2sh#1, or sh#2 together with GFP. Next, neurons were treated with 20 nM rapamycin or DMSO (control), fixed, and immunostained for P-S6. Representative images, results of analysis of P-S6 intensity, and neuron soma area. Scale bar: 25 μm. Plots represent mean +/−SEM. **p < 0.01, ***p < 0.001 in Kruskal-Wallis with Dunn’s post-hoc test. Sample sizes for experimental groups are provided in Supplementary materials and methods (Additional file 1). For additional results of TSC2sh validation, see Additional file 3: Figure S1. **b** Results of analysis of neuronal soma area from screening experiments. Each point represents mean +/− SEM obtained from two independent experiments. For more details see Additional file 2: Table S1 and Additional file 3: Table S2. **c** Western blot analysis of GCLC level in rat cortical neurons 4 days after nucleofection with empty pSuper vector, GCLCsh-mix used in the screening experiments or individual GCLCsh#1, sh#2, and sh#3. α-tubulin is shown as a loading control. **d** Representative images of rat cortical neurons after TSC2 knockdown with use of 2 different shRNAs and simultaneous GCLC knockdown with use of mixed shRNAs (as in the screen) or 2 individual shRNAs. GFP-encoding plasmid was co-transfected for visualization of modified neurons. Scale bar: 50 μm. **e** Neuronal soma area of rat cortical neurons after TSC2 and GCLC knockdown. Plot represents mean +/−SEM. ns- not significant, *p < 0.05, **p < 0.01, ***p < 0.001 in Kruskal-Wallis with Dunn’s post-hoc test. Sample sizes for experimental groups are provided in Supplementary materials and methods (Additional file 1)

One characteristic feature of TSC is the presence of enlarged dysmorphic neurons and giant cells in cortical tubers [13]. This aberrant cell size increase is thought to be due to loss of either *TSC1* or *TSC2* and mTORC1 overactivation [12, 39]. To enable screening for the genes and proteins required for the mTORC1-dependent cell size increase, we first modeled aberrant neuronal growth *in vitro*. Utilizing plasmid-based shRNA expression [3], we efficiently depleted cultured rat cortical neurons of *TSC2* (Additional file 3: Figure S1a) using either one of two shRNAs (TSC2sh#1, TSC2sh#2). When introduced to neurons at Day 6 *in vitro* (DIV6), these shRNAs effectively increased (i) levels of ribosomal S6 protein phosphorylated at Ser235/236 (P-S6) and (ii) neuronal soma size (Fig. 1a). Both observations are consistent with the data from TSC patients [36], TSC-model mouse brain tissue [12, 43], and *in vitro* TSC models [28, 39]. Importantly, both the increase in P-S6 levels and cell soma size were blocked by treatment with an mTOR inhibitor (20 nM rapamycin) (Fig. 1a). The specificity of both TSC2sh was confirmed by “rescue” experiments, in which neuronal overgrowth was prevented by co-transfecting neurons with TSC2sh and a plasmid encoding TSC2 mRNA resistant to shRNA (Additional file 3: Figure S1b and S1c).

Having validated our *in vitro* model of mTOR-dependent neuronal hypertrophy, we performed screening experiments to identify genes and proteins necessary for the aberrant cell size increase. To this end, we used a library of shRNAs designed to target mRNAs encoding 147 proteins linked to mTOR signaling pathway or TSC pathology in past studies (see Supplementary materials and methods (Additional file 1) for detailed library description and the list of targeted genes and Additional file 2: Table S1 for shRNAs sequences). shRNAs targeting given mRNA were transfected to cortical neurons as pools to ensure effective knockdown. The pools of shRNAs were co-transfected to DIV6 neurons with TSC2sh#1 and plasmid encoding GFP, for visualization of transfected cell morphology. As a control, we used neurons transfected with (i) empty pSuper, (ii) pSuper and TSC2sh#1, and (iii) pSuper and TSC2sh#1, treated with 20 nM rapamycin. Analysis of the cell somas of transfected neurons 4 days post transfection revealed that the majority of tested shRNAs had no effect on TSC2sh#1-induced hypertrophy. However, 30 shRNA pools caused a significant decrease in the neuronal soma area of TSC2-depleted cells (Additional file 3: Figure S1b and Table S2). Twenty-three of these genes were confirmed in follow-up experiments using TSC2sh#2 instead of TSC2#sh1 (Additional file 3: Table S2).

### TSC-related neuronal hypertrophy depends on GCLC

We subsequently decided to focus on GCLC, a first rate limiting enzyme of glutathione synthesis that was previously identified in a yeast study as potentially linked to TOR signaling [5]. GCLC plays a crucial role in protecting cells from oxidative stress and damage caused by reactive oxygen species (ROS). There is also evidence indicating increased cellular stress and ROS levels in TSC pathology [28].

To verify the results of screening experiments, we tested the specificity of GCLC shRNAs and its phenotypic effects. Indeed, all three GCLCsh individually and as a pool efficiently reduced levels of GCLC in nucleofected cortical neurons cultured *in vitro* (Fig. 1c). We next tested effects of single shRNAs on the phenotype of the model hypertrophic neurons and confirmed that both GCLCsh#1 and GCLCsh#3, similar to the GCLCsh pool, reversed the hypertrophic phenotype induced by TSC2 depletion with either TSC2sh#1 or TSC2sh#2 (Fig. 1d and 1e). GCLCsh#2 appeared to have non-specific toxic effects when transfected to DIV6 neurons, and therefore was not studied further.

**Fig. 2:**
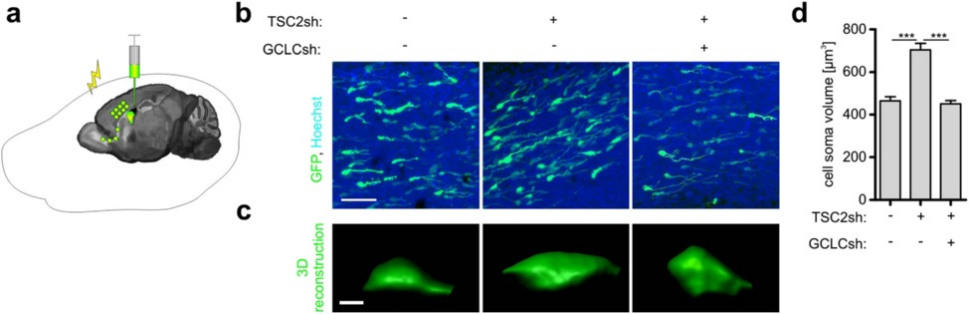
GCLCsh blocks aberrant growth in TSC in vivo model. **a** Scheme of in vivo electroporation. **b** Representative images of cells in rostral migratory stream obtained in in vivo experiments. Newborn rats were electroporated with pSuper vector or TSC2sh#2 together with pSuper or GCLCsh#1. Plasmid encoding GFP was used to visualize modified cells. Scale bar: 50 μm. **c** Exemplary 3D reconstructions of GFP-positive cells in rostral migratory stream. Scale bar: 3 μm. **d** Quantification of cell soma volume of cells migrating in the rostral migratory stream. Plot represents mean +/−SEM. ***p < 0.001 in Kruskal-Wallis with Dunn’s post-hoc test. Sample sizes for experimental groups are provided in Supplementary materials and methods (Additional file 1)

We next examined whether GCLC is important for the hypertrophy of TSC2-depleted neurons in vivo. For this purpose, we performed in vivo electroporation of shRNAs encoding plasmids into proliferating neuronal precursors in the subventricular zone (SVZ) of newly born rodents (Fig. 2a), as described previously [8, 22]. Six days after electroporation, the TSC2sh#2-electroporated neuroprogenitors in the rostral migratory stream had a significantly higher volume when compared to the cells of control rats (Fig. 2b-2d). Consistent with our observations from cultured neurons, co-electroporation of GCLCsh with TSC2sh blocked the size increase of TSC2-deficient cells.

**Fig. 3:**
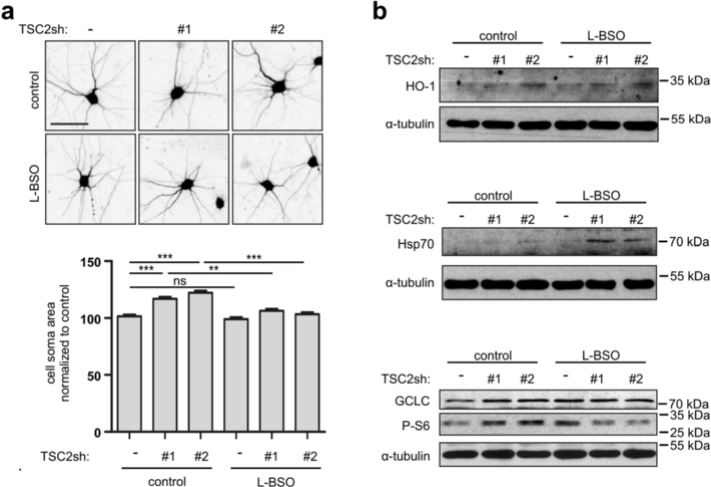
GCLC inhibitor L-BSO blocks aberrant growth, induces stress response and inhibits mTORC1 in TSC2 depleted neurons. **a** Representative images and neuron soma area of TSC2-depleted and control neurons treated with 10 μM L-BSO. Scale bar: 50 μm. Plot represents mean +/−SEM. ns- not significant, **p < 0.01, ***p < 0.001 in Kruskal-Wallis with Dunn’s post-hoc test. Sample sizes for experimental groups are provided in Supplementary materials and methods (Additional file 1). **b** Western blot analysis of HO-1, Hsp70, GCLC and mTORC1 activity marker, P-S6, levels in TSC2-depleted and control neurons after L-BSO treatment. α-tubulin is shown as a loading control

Furthermore, we verified the importance of GCLC for aberrant growth of TSC2-defficient neurons using a specific inhibitor of GCLC catalytic activity, L-Buthionine-sulfoximine (L-BSO) [14]. Neuronal soma enlargement was fully blocked by the addition of L-BSO (10 μM) to the cultured neurons transfected with TSC2sh (either #1 or #2), supporting our finding that GCLC is necessary for TSC-related aberrant growth (Fig. 3a).

### GCLC inhibition increases cellular stress and reduces mTOR activation in TSC2-depleted neurons

Previous work showed that knocking out or down TSC2 elevates cellular stress [28, 32] and eventually leads to apoptotic death in neurons [28]. Yet, TSC-lacking brain cells survive in both mouse models and TSC patients’ brain lesions, suggesting their adaptation to such stress. We thus hypothesized that if GCLC contributes to this adaptation, blocking its activity in TSC2-deficient cells should perturb the redox balance. Consistent with previous findings, TSC2 knockdown neurons showed higher levels of oxidative stress markers—heme oxygenase 1 (HO-1) and heat shock protein 70 (Hsp70)—compared to control cells (Fig. 3b). Importantly, inhibiting GCLC activity with L-BSO further increased HO-1 and Hsp70 in TSC2-deficient cells but did not lead to visible induction of those markers in control neurons (Fig. 3b). We also note that GCLC level is higher in TSC2-depleted cells and, in contrast to control cells, cannot be further increased upon L-BSO treatment (Fig. 3b). As GCLC mitigates oxidative stress, its elevated expression is likely an adaptive response to an increase in ROS in TSC2 knockdown neurons. Inhibition of GCLC seems to disturb this adaptation, increases cellular stress, and prevents aberrant cell size increase. Importantly, GCLC inhibition decreases mTORC1 activity reflected by P-S6 levels in TSC2-depleted neurons but not in control cells (Fig. 3b), which could explain the inhibition of aberrant growth.

### TSC patients’ brain lesions show increased levels of stress markers and GCLC

**Fig. 4:**
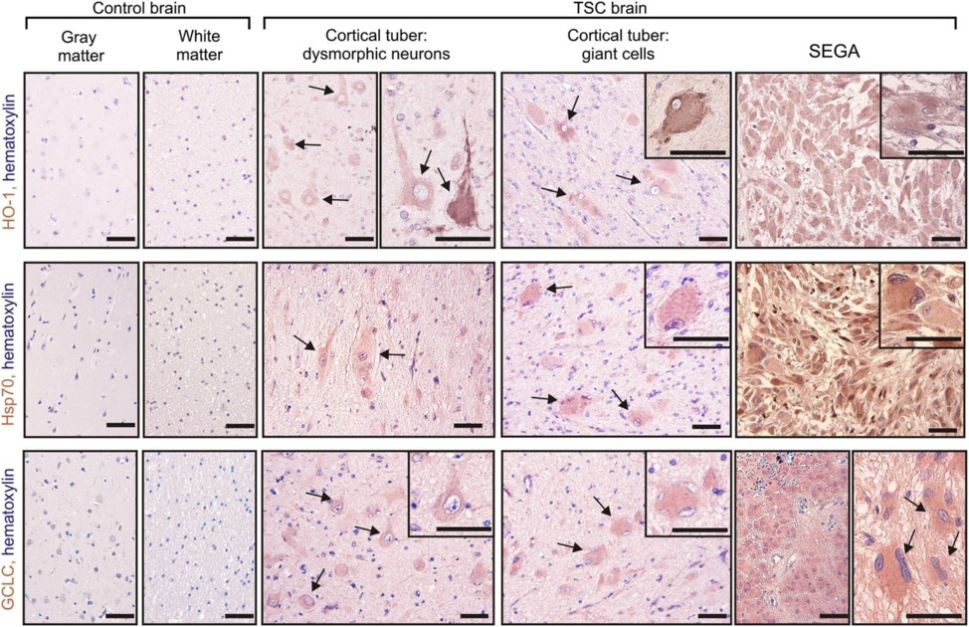
Patients’ brain lesions show markers of oxidative stress and GCLC expression. Representative images of immunohistochemical staining of Heme oxygenase 1 (HO-1), Heat shock protein 70 (Hsp70), glutamate-cysteine ligase catalytic subunit (GCLC) in control brain sections (gray and white matter) and TSC patients’ brain sections (cortical tubers and SEGAs). Arrows point to dysmorphic neurons, giant cells, and enlarged SEGA cells. Cell nuclei are stained with hematoxylin. Insets show higher magnification. Scale bar: 80 μm. For additional controls, see Additional file 3: Figure S2

Assuming that GCLC-dependent oxidative stress adaptation is necessary to develop TSC-related cellular hypertrophy, we should observe increased stress markers and GCLC also in cortical tubers in patients’ tissue. To test this, we immunostained brain sections from individuals affected with TSC for HO-1 and Hsp70 as well as for GCLC (Fig. 4, see also Additional file 3: Figure S2 for additional controls). Consistent with the conclusions from published data [28] and our *in vitro* studies, we observed the presence of those proteins in dysmorphic neurons and the giant cells of cortical tubers, but not in control brain tissue from individuals not affected with TSC, nor in surrounding normal-appearing cells (Fig. 4). Intriguingly, similar results were obtained for enlarged SEGA cells as compared to control brain tissue (Fig. 4), suggesting that the hypertrophy of cells in different types of brain TSC lesions may depend on GCLC.

### GCLC inhibition affects SEGA-derived cells growth, proliferation, and survival

**Fig. 5:**
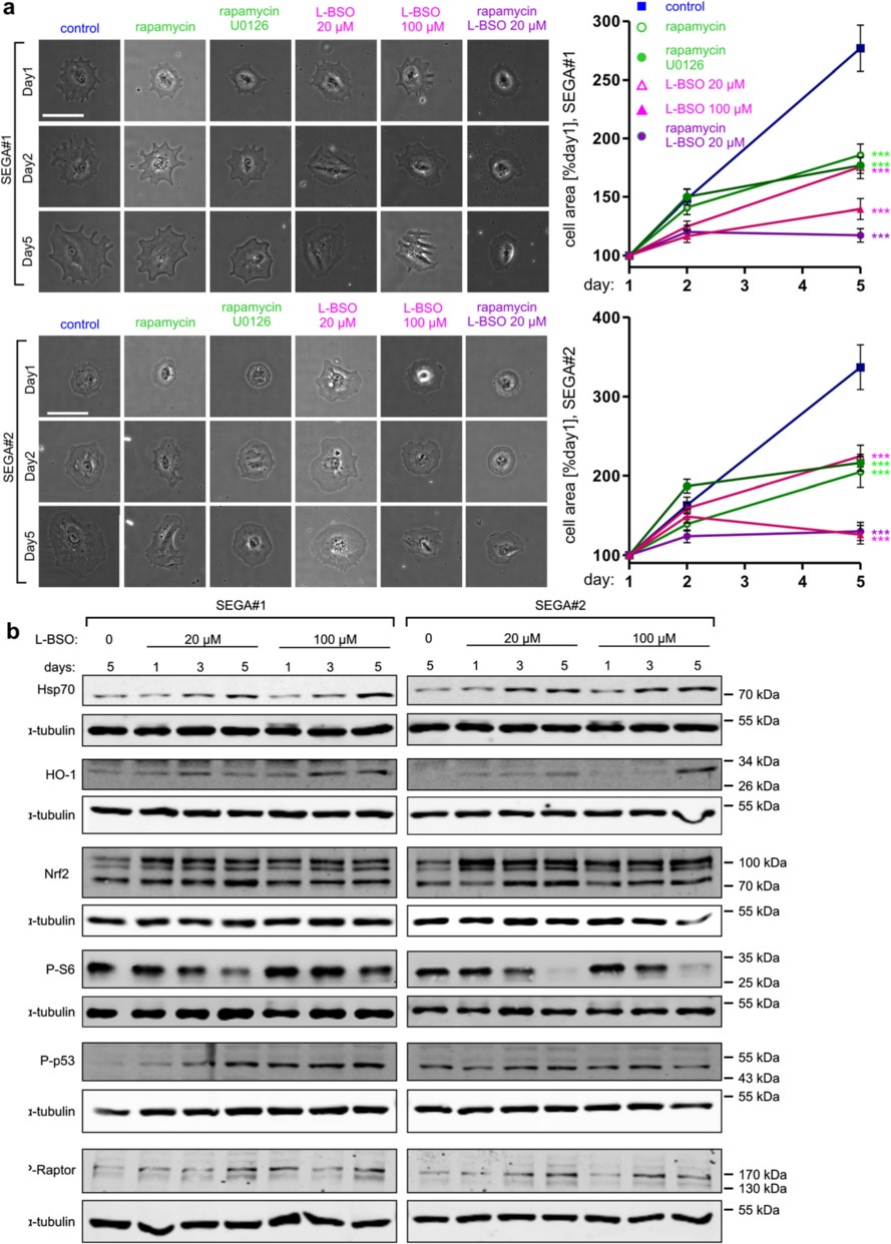
GCLC inhibition blocks growth of SEGA-derived cells and causes cellular stress, and mTORC1 inhibition. **a** Representative images (left) and results of cell surface area analysis (right) of SEGA-derived cells imaged 3 times during a 5 day-treatment. SEGA#1 and SEGA#2-derived cells were treated with 20 nM rapamycin, 20 nM rapamycin with 20 μM U0126 or 20 μM L-BSO, and 20 or 100 μM L-BSO. Scale bar: 100 μm. The plots represent mean +/− SEM from two independent experiments. ***p < 0.001 in two-way ANOVA compared to control treated with DMSO. Sample sizes for experimental groups are provided in Supplementary materials and methods (Additional file 1). **b** Western blot analysis of stress markers (Hsp70, HO-1), Nrf2, mTORC1 activity marker P-S6, genotoxic stress marker P-p53 and P-Raptor in SEGA-derived cells lysed after a 1-, 3- or 5-day treatment with 20 or 100 μM L-BSO. α-tubulin is shown as a loading control

Since SEGAs are constantly growing lesions that often require surgical resection or treatment with mTORC1 inhibitors, we examined the effects of GCLC inhibition on the growth of cultured SEGA cells derived at the time of surgical resection from two TSC patients (Additional file 3: Figure S3a). To circumvent the heterogeneity of the cells observed in these cultures, we used live imaging of SEGA-derived cells during treatment with L-BSO (Fig. 5). This approach enabled reliable quantification of cell size increase in comparison to initial cell area and allowed us to exclude from analysis cells that divided or died over this time.

L-BSO added to the culture media, at either 20 or 100 μM, significantly slowed down the growth of two SEGA-derived lines (Fig. 5a). A similar effect, although less potent than that of a higher L-BSO dose, was obtained upon rapamycin or rapamycin/U0126 (extracellular signal-regulated kinase inhibitor) treatment, as described previously, on cultured SEGA-derived cells [42]. On the other hand, lower dose of L-BSO combined with rapamycin slowed down growth of tested cells similar to the 100 μM L-BSO (Fig 5a).

Of note, L-BSO treatment in both cell lines also induced expression of cellular stress markers Hsp70 and HO-1, as well as Nrf2, a transcription factor coordinating expression of several oxidative stress-inducible genes, including HO-1 [16]. Moreover, as in case of TSC2-depleted neurons, L-BSO treatment lead to decrease in P-S6 levels (Fig. 5b; see also Additional file 3: Figure S4 for WB results quantification). It should be noted however, that Ser 235/236 S6 phosphorylation could also decrease due to a drop in extracellular-signal-regulated kinases (ERKs) activity in response to L-BSO. Therefore, we checked levels of P-ERK1/2 (Thr 202/Tyr 204) in L-BSO-treated cells and could not detect any changes over time (Additional file 3: Figure S5). In contrast, we noticed a reduced phosphorylation of Thr 389 of p70S6K, a direct mTORC1 target (Additional file 3: Figure S5). This supports our initial conclusion that L-BSO gradually decreases mTORC1 activity in SEGA-derived cells. We hypothesized that the observed mTORC1 inhibition is an effect of ROS-induced DNA damage. Indeed, supporting such mechanism, SEGA-derived cells treated with L-BSO show progressive increase in p53 phosphorylation (P-p53, Ser15), which is a hallmark of DNA damage response [37] (Fig. 5b; see also Additional file 3: Figure S4 for WB results quantification). We also observed parallel increase in inhibitory phosphorylation of Raptor (P-Raptor, Ser792), a key component of mTORC1 (Fig. 5b; see also Additional file 3: Figure S4 for WB results quantification). P-p53 has been shown to activate AMPK [7, 10], which in turn phosphorylates Raptor and inhibits mTORC1 [15].

**Fig. 6:**
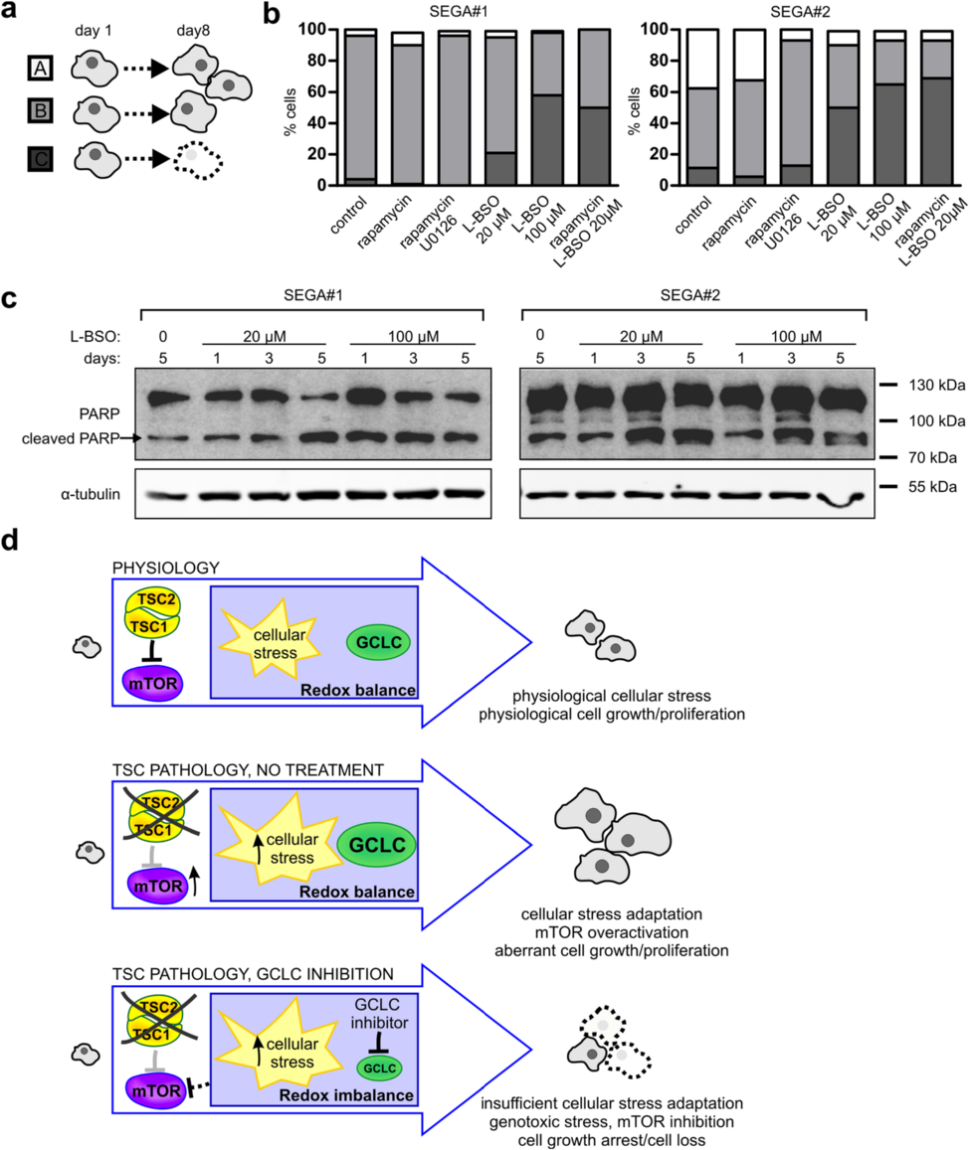
GCLC inhibition causes death of SEGA-derived cells. **a** Schematic representation of SEGA-derived cell classes after an 8-day treatment. **b** Percentage of cells classified to class A, B, or C after treatment with 20 nM rapamycin, 20 nM rapamycin with 20 μM U0126 or 20 μM L-BSO, and 20 or 100 μM L-BSO. SEGA#1 and SEGA#2-derived cells were imaged 4 times during the treatment. Next, the fate of each cell was followed and classes were assigned. Sample sizes for experimental groups are provided in Supplementary materials and methods (Additional file 1). For additional information, see also Additional file 3: Figure S3. **c** Western blot analysis of PARP (full length and cleaved, upper and lower band, respectively) in SEGA-derived cells lysed after a 1-, 3- or 5-day treatment with 20 or 100 μM L-BSO. α-tubulin is shown as a loading control. **d** Proposed model of GCLC contribution to TSC-related tumors development

It is well established that genotoxic stress may affect cell proliferation and survival. To address this issue, we examined the effects of longer periods of L-BSO treatment on SEGA-derived cell fates. For this purpose, we performed live imaging for a total of 8 days. For quantification, we divided cells present at day 1 into 3 classes: cells that finally gave rise to one cell by Day 8 (in most cases neither divided nor died; class A), cells that gave rise to two or more cells (in most cases divided at least once; class B), and those that died (class C) (Fig. 6a, see also Additional file 3: Figure S3b and S3c for a more detailed description of classes). In response to prolonged L-BSO treatment, the two SEGA-derived cell lines both showed marked reduction in cell division, and increased numbers of cells were lost. These effects were more dramatic compared to treatment with either rapamycin alone or combined with U0126 (Fig. 6b). However, 20 μM L-BSO-rapamycin combined treatment gave effects comparable to higher L-BSO dose (Fig. 6b). Moreover, L-BSO treatment induced cleavage of Poly(ADP-ribose) polymerase 1 (PARP-1; Fig. 6c), confirming SEGA-derived cells death.

In conclusion, we propose that mechanisms underlying L-BSO effects on TSC cells involve induction of redox imbalance, oxidative DNA damage and subsequent increase in P-p53. These events lead consequently to growth arrest due to AMPK-P-Raptor-dependent inhibition of mTORC1 and genotoxic cell death.

## Discussion

Our data clearly established the requirement of GCLC for the development of TSC-related phenotype. Findings from *in vitro* and in vivo models, as well as study of SEGA-derived cell growth, point to GCLC inhibition as a potential strategy in TSC treatment. Moreover, we observed substantial induction of cellular stress markers and GCLC in TSC patients’ brain tubers, which supports the clinical relevance of our findings.

The primary finding of our study is that GCLC contributes to the hypertrophy and survival of TSC2-deficient cells. We describe increased cellular stress marker expression and mTORC1 inhibition upon L-BSO treatment, both in case of TSC2 neuronal knockdown and SEGA-derived cells. Mechanisms of this inhibition most likely involve genotoxic stress-dependent p53 phosphorylation and subsequent inactivation of mTORC1 due to AMPK-dependent phosphorylation of Raptor. Importantly, mTORC1 inhibition upon L-BSO treatment was observed exclusively in TSC2-deficient cells, which may have important clinical implications.

Our data suggest that GCLC provides protection against elevated ROS level in TSC2-deficient cells. Redox adaptation has also been described for various cancer cells [40]. The signatures of this phenomenon include induction of ROS scavenging enzymes expression and increased sensitivity to the depletion of glutathione. In a similar vein, it has been demonstrated that expression of Hif1α, known to be a part of antioxidant defense in response to ROS, is highly elevated in TSC1-deficient cells [9, 33]. Herein, we provide evidence for increased GCLC levels, suggesting that this ROS scavenging systems is also upregulated. Targeting redox homeostasis or increasing cellular stress is considered a supplemental therapy for many cancers [40]. Previously, increasing ER stress proved efficient in boosting selective cell death in TSC-deficient cells, and was therefore suggested as a potential therapy [28, 32]. In this paper, we demonstrate that another approach, namely, targeting the synthesis of ROS scavengers, is effective in preventing cellular hypertrophy as well as inducing the loss of cells with disturbed TSC, likely due to mTORC1 inhibition and genotoxic DNA damage, respectively. Of note, unlike ER stress inductor tunicamycin, L-BSO has some synergistic effects with rapamycin, which might be beneficial for TSC pharmacotherapy. Our observations are also in line with recent work from Blenis lab, showing that combined ER stress induction and glutathione depletion leads to death of TSC2 knockout fibroblasts cultured *in vitro* and regression of TSC2-deficient xenograft tumors development [23]. This finding strongly corroborates our conclusion that inhibition of GCLC can be considered as a new, strategy for treatment of different types of TSC-related tumors. Moreover synergistic effects of GCLC inhibition with rapamycin treatment would allow combined therapy.

Our results thus suggest that GCLC inhibition could be a novel strategy in the pharmacotherapy of TSC-related brain tubers. However, the consequences of systemic GCLC inhibition remain underinvestigated. For example, BSO toxicity was shown in rats [17]. Nevertheless, BSO was tested in phase I clinical trial for treatment of refractory malignancies with the conclusion that it could be safely administered to patients [1]. Therefore, in our opinion, this line of research is promising, since currently available pharmacotherapy of TSC-related brain tubers, based on mTOR inhibitors, has important limitations. Although certainly beneficial for patients, the treatment does not cause persistent eradication of SEGA tubers and therefore needs to be continued for longer periods of time [31]. In addition, although in our study rapamycin treatment reduces SEGA-derived cell growth, it does not lead to cell loss. On the contrary, L-BSO alone or at lower doses in combination with rapamycin (not shown) caused SEGA-derived cell death. If the same results are obtained in patient treatment, pharmacotherapy could perhaps be shortened, which would be a great advantage over mTOR inhibitors, known to severely impair vital cellular processes also in healthy cells. In summary, we identified GCLC as an important player in TSC-related hypertrophy and a potential target for the future pharmacotherapy of SEGA.

## Conclusions

In this study, we aimed to provide new potential drug targets in TSC pharmacotherapy by identifying proteins crucial for the pathological overgrowth typical of TSC. Consequently, using in vitro and in vivo rodent models, as well as SEGA-derived cells cultured in vitro we describe GCLC as a part of redox adaptation in TSC, needed for overgrowth and survival of mutant cells. Inhibition of GCLC with L-BSO was able to prevent TSC related cell hypertrophy and induce mutant cell death, suggesting GCLC inhibition as a potential strategy in TSC treatment. We propose that mechanisms underlying effects of L-BSO on TSC cells involve induction of redox imbalance, oxidative DNA damage and subsequent increase in P-p53. These events lead consequently to growth arrest due to AMPK-P-Raptor-dependent inhibition of mTORC1 and genotoxic cell death.

## Additional files

Additional file 1: **Supplementary materials and methods.** (PDF 153 kb)

Additional file 2: **Table S1.** (XLS 163 kb)

Additional file 3: **Supplementary Figures and Table S2.** (PDF 504 kb)
