# Supplementary material for: Tuberous sclerosis complex neuropathology requires glutamate-cysteine ligase
Source: Acta Neuropathol Commun. 2015 Jul 30;3:48. doi: 10.1186/s40478-015-0225-z (PMC4518593; doi:10.1186/s40478-015-0225-z)
Supplement: Supplementary file 1 — Supplementary materials and methods. (PDF 153 kb) [file 40478_2015_225_MOESM1_ESM.pdf]

Online Resource 1

Title: Tuberous sclerosis complex neuropathology requires glutamate-cysteine ligase
Malik *et al.*

Supplementary materials and methods

Drugs and antibodies

The following inhibitors were purchased from commercial sources: Rapamycin (Calbiochem, La Jolla, CA), L-Buthionine-sulfoximine (L-BSO; Sigma, St Louis, MO), U0126 (Promega, Madison, WI). Primary antibodies are listed in the table below.

Antibodies used for the studies

Antigen	Manufacturer	Cat. number	Host	Application	Dilution
TSC2	Cell Signaling, Danvers, MA	4308	Rabbit	WB	1:1000
TSC1	Abcam, Cambridge, UK	Ab21632	Rabbit	WB	1:1000
P-S6 (Ser235/236)	Cell Signaling Danvers, MA	4858	Rabbit	IF, WB	1:150, 1:1000
P-p70S6K (Thr389)	Cell Signaling Danvers, MA	9206	Mouse	WB	1:1000
α-tubulin	Sigma, St Louis, MO	T5168	Mouse	WB	1:5000
GFP	Roche, Basel, Switzerland	11814460001	Mouse	WB	1:5000
GFP	MBL	598	Rabbit	IF (rat brain sections)	1:250
β-galactosidase	Promega, Madison, WI	Z3871	Mouse	WB	1:1000
GCLC	Abcam, Cambridge, UK	ab55435	Mouse	WB	1:500
GCLC	Aviva Systems Biology, San Diego, CA	ARP54576_P050	Rabbit	IHC (human brain sections)	1:50
GCLC	Santa Cruz Biotechnology, Dallas, TX	SC-22755	Rabbit	IHC (human brain sections)	1:100
HO-1	Enzo Life Sciences, Farmingdale, NY	ADI-SPA-896	Rabbit	WB	1:500
HO-1	Abcam, Cambridge, MA, USA	ab13243	Rabbit	IHC (human brain sections)	1:100
Hsp70	Santa Cruz Biotechnology CA, USA	sc-33575	Rabbit	IHC (human brain sections)	1:100

Hsp70	NovoCastra, Newcastle upon Tyne, UK	NCL-HSP70	Mouse	IHC (human brain sections)	1:20
Hsp70	Enzo Life Sciences, Farmingdale, NY	ADI-SPA-812	Rabbit	WB	1:5000
Nrf2	Santa Cruz Biotechnology CA, USA	SC-13032	Rabbit	WB	1:500
P-Raptor (Ser792)	Cell Signaling Danvers, MA	2083	Rabbit	WB	1:500
P-p53 (Ser15)	Cell Signaling, Danvers, MA	9284	Rabbit	WB	1:1000
PARP	Cell Signaling, Danvers, MA	9532	Rabbit	WB	1:1000
P-ERK1/2 (Thr201/Tyr204)	Cell Signaling, Danvers, MA	9101	Rabbit	WB	1:1000

IF: immunofluorescence; IHC: immunohistochemistry; WB: Western blot

Secondary anti-rabbit and anti-mouse antibodies conjugated to horseradish peroxidase (HRP) (Jackson ImmunoResearch, West Grove, PA, 1:10000)) or IRDyes (LI-COR Biosciences, Lincoln, NE, 1:5000) were used for WB. Donkey anti-rabbit and anti-mouse secondary antibodies conjugated to Alexa Fluor 568 and Alexa Fluor 488 (Invitrogen, Eugene, OR; 1:200), respectively, were used for IF (both on cultured cells and brain sections).

DNA constructs

mRNA sequences targeted by most frequently used shRNAs were as follows: TSC2sh#1: GGAUAAAGACCAUCAGGUU, TSC2sh#2: GGUGAAGAGAGCCGUAUCACA, GCLCsh#1: AGCAUAGACACCAUCAUCA, GCLCsh#2: GCAUCUAAGUCCCUCUUCU, GCLCsh#3: GCAAACCAUCCUGACUACA. The coding sequence of TSC2 was amplified from TSC2-pBluescript (generous gift from Dr. T. Kobayashi) and the product was cloned to SalI/XbaI sites of pEGFP-C1 (Clontech, Mountain View, CA). To obtain shRNA-resistant mutants for rescue experiments, we performed site-directed mutagenesis using the following primers: CTCCCACATCCCCGAAGACAAGGACCACCAAGTCCGAAAGCTGGCTACC (TSC2*1) and TGGTCAGCTCCGGGTCAAAAGGGCAGTCTCCCAGTCCCACCTGAAG (TSC2*2).

shRNA library

The majority of targets for shRNA library was selected based on data from yeast studies, in which the growth of mutant strains in the presence of rapamycin was investigated (Butcher *et al.*, 2006; Chan *et al.*, 2000; Xie *et al.*, 2005). A second large group of targets came from microarray studies focusing on transcriptional changes during activation or inhibition of mTOR signaling (Guertin *et al.*, 2006). The third group of targets came from the mRNA profiling of TSC KO mice or SEGA-derived cell lines (Onda *et al.*, 2002; Tyburczy *et al.*, 2010). Other proteins targeted by the library were locally translated in neurons depending on mTOR activity (Schratt *et al.*, 2004) or were previously described as mTOR targets (Caron *et al.*, 2010). shRNA library targets are listed in the table below.

Targets for shRNA library

Gene name	Rat gene/protein number in a database		Human gene number in Ensembl database
	Uniprot/EMBL	Ensembl	
Actb	EMBLX03672	ENSRNOG00000034254	ENSG00000075624
Adfp	EMBL M93275	ENSRNOG00000007060	ENSG00000147872
Agxt	Uniprot P09139	ENSRNOG00000023856	ENSG00000172482
ALG5	Uniprot Q9Y673	ENSRNOG00000013485	ENSG00000120697
Anxa1	EMBL X07486	ENSRNOG00000017469	ENSG00000135046
Anxa3	EMBL AJ001633	ENSRNOG00000002045	ENSG00000138772
Anxa6	Uniprot Q6IMZ3	ENSRNOG00000010668	ENSG00000197043
Ap2b1	Uniprot P62944	ENSRNOG00000009988	ENSG00000006125
Arfgap1	Uniprot Q62848	ENSRNOG00000043150	ENSG00000101199
Arfrp1	Uniprot Q63055	ENSRNOG00000013992	ENSG00000101246
ASH2L	Uniprot Q9UBL3	ENSRNOG00000014875	ENSG00000129691
Asp1	EMBL AF216310	ENSRNOG00000001953	ENSG00000182240
Ass	EMBL M31690	ENSRNOG00000008837	ENSG00000130707
ATBD1C	-	ENSRNOG00000001278	ENSG00000111231
ATP6V1H	Uniprot Q9UI12	ENSRNOG00000030862	ENSG00000047249
ATPbc1	EMBL AF287263	ENSRNOG00000018126	ENSG00000165029
Atpbd3	-	ENSRNOG00000018334	ENSG00000142544

Bgt2	EMBL M64292	ENSRNOG00000003300	ENSG00000159388
BOP1	Uniprot Q14137	ENSRNOG00000021773	ENSG00000170727
Brdt	Uniprot Q6AYL3	ENSRNOG00000002073	ENSG00000137948
Cadps2	-	ENSRNOG00000007636	ENSG00000081803
Cckbr	Uniprot P30553	ENSRNOG00000017679	ENSG00000110148
CCNA2	Uniprot P20248	ENSRNOG00000015423	ENSG00000145386
Ccnd1	Uniprot P39948	ENSRNOG00000020918	ENSG00000110092
CDKN1A	Uniprot P38936	ENSRNOG00000000521	ENSG00000124762
Chga	Uniprot P10354	ENSRNOG00000007912	ENSG00000100604
Chrna2	Uniprot P12389	ENSRNOG00000017424	ENSG00000120903
CNDP1	Uniprot Q96KN2	ENSRNOG00000027739	ENSG00000150656
CNDP2	Uniprot Q96KP4	ENSRNOG00000015591	ENSG00000133313
Cntnap1	Uniprot P97846	ENSRNOG00000020277	ENSG00000108797
Coq2	Uniprot Q499N4	ENSRNOG00000002194	ENSG00000173085
Cryab	EMBL M63170	ENSRNOG00000010524	ENSG00000109846
cyr61	EMBL M32490	ENSRNOG00000014350	ENSG00000142871
Ddr1	Uniprot Q63474	ENSRNOG00000000830	ENSG00000204580
DDX18	Uniprot Q9NVP1	ENSRNOG00000025430	ENSG00000088205
DIAPH1	Uniprot O60610	ENSRNOG00000019688	ENSG00000131504
Dpf1	Uniprot P56163	ENSRNOG00000020687	ENSG00000011332
Dusp1	EMBL S64851	ENSRNOG00000003977	ENSG00000120129
Egr1	Uniprot P08154	ENSRNOG00000019422	ENSG00000120738
FATh2	Uniprot O88277	ENSRNOG00000012575	ENSG00000086570
Fbp2	Uniprot Q9Z1N1	ENSRNOG00000017637	ENSG00000130957
Fgf10	Uniprot P70492	ENSRNOG00000012278	ENSG00000070193
Fkbp1a	Uniprot Q62658	ENSRNOG00000008822	ENSG00000088832
Furin	Uniprot P23377	ENSRNOG00000011352	ENSG00000140564
Gabrg2	Uniprot P18508	ENSRNOG00000003241	ENSG00000113327
GARS	Uniprot P41250	ENSRNOG00000011052	ENSG00000106105
Gclc	Uniprot P19468	ENSRNOG00000006302	ENSG00000001084
Ghsr	Uniprot O08725	ENSRNOG00000024119	ENSG00000121853
Gmfb	Uniprot Q63228	ENSRNOG00000010061	ENSG00000197045
Gnb3	Uniprot P52287	ENSRNOG00000015541	ENSG00000111664

Gosr1	Uniprot Q62931	ENSRNOG00000003971	ENSG00000108587
GPNMB	EMBL AJ251685	ENSRNOG00000008816	ENSG00000136235
Gpx4	EMBL AF045769	ENSRNOG00000013604	ENSG00000167468
Grip1	-	ENSRNOG00000004013	ENSG00000155974
Grk1	Uniprot Q63651	ENSRNOG00000018430	ENSG00000185974
Gsl	EMBL J04953	ENSRNOG00000018991	ENSG00000148180
HEATR1	Uniprot Q9H583	ENSRNOG00000021686	ENSG00000119285
HGS	Uniprot O14964	-	ENSG00000185359
HSPA4	Uniprot P34932	ENSRNOG00000016596	ENSG00000170606
Hspa4l	Uniprot O95757	ENSRNOG00000010819	ENSG00000164070
Itm2b	EMBL U76253	ENSRNOG00000016271	ENSG00000136156
Jundp2	NCBI U53449	ENSRNOG00000008224	ENSG00000140044
Kcnc3	Uniprot Q01956	ENSRNOG00000019959	ENSG00000131398
KCNG1	Uniprot Q9UIX4	ENSRNOG00000012144	ENSG00000026559
KIAA0804	-	ENSRNOG00000001764	ENSG00000156931
KIAA1189	-	ENSRNOG00000021472	ENSG00000136541
KTI12	Uniprot Q5I0L7	-	ENSG00000198841
Limk1	Uniprot P53669	ENSRNOG00000001470	ENSG00000106683
Mapk3	Uniprot P21708	ENSRNOG00000019601	ENSG00000102882
MCM4	Uniprot P33991	ENSRNOG00000001833	ENSG00000104738
MLC2	NCBI M11851	-	ENSG00000111245
MOCS3	Uniprot O95396	ENSRNOG00000025067	ENSG00000124217
MRPL27	Uniprot Q9P0M9	ENSRNOG00000003724	ENSG00000108826
Nat10	Uniprot Q9H0A0	ENSRNOG00000008663	ENSG00000135372
Ndrg1	EMBL U60593	ENSRNOG00000007393	ENSG00000104419
Neurod1	Uniprot Q64289	ENSRNOG00000005609	ENSG00000162992
NOP14	Uniprot P78316	ENSRNOG00000012147	ENSG00000087269
Nptx1	EMBL U62021	ENSRNOG00000003741	ENSG00000171246
NptxR	EMBL AF318076	ENSRNOG00000016156	ENSG00000221890
Nrg2	Uniprot O35569	ENSRNOG00000019093	ENSG00000158458
Nsun2	-	ENSRNOG00000017254	ENSG00000037474
Ormdl3	Uniprot Q6QI25	ENSRNOG00000030445	ENSG00000172057
Perld1	Uniprot B1WBW5	-	ENSG00000161395

Pgap1	-	ENSRNOG00000013388	ENSG00000197121
Pik3c2g	Uniprot O70173	ENSRNOG00000034228	ENSG00000139144
Pla2g7	EMBL U34277	ENSRNOG00000025691	ENSG00000146070
Plk2	Uniprot Q9R012	ENSRNOG00000011951	ENSG00000145632
Plk3	Uniprot Q9R011	ENSRNOG00000018484	ENSG00000173846
Plod2	EMBL AF080572	ENSRNOG00000030183	ENSG00000152952
Polr1a	Uniprot O54889	ENSRNOG00000009545	ENSG00000068654
Polr1b	Uniprot O54888	ENSRNOG00000018349	ENSG00000125630
POLR2B	Uniprot P30876	ENSRNOG00000024779	ENSG00000047315
POLR3B	Uniprot Q9NW08	ENSRNOG00000007432	ENSG00000013503
PPP2R4	Uniprot Q15257	ENSRNOG00000018457	ENSG00000119383
Ppp6c	Uniprot Q64620	ENSRNOG00000015145	ENSG00000119414
Prmt3	Uniprot O70467	ENSRNOG00000014829	ENSG00000185238
PSCD1	Uniprot P97694	ENSRNOG00000043381	ENSG00000108669
Psen1	Uniprot P97887	ENSRNOG00000009110	ENSG00000080815
Pvalb	Uniprot P02625	ENSRNOG00000006471	ENSG00000100362
RAB33B	Uniprot Q9H082	ENSRNOG00000013035	ENSG00000172007
Rabep2	NCBI U34932	-	ENSG00000177548
RAPTOR	Uniprot Q8N122	ENSRNOG00000003821	ENSG00000141564
RATBSP	Uniprot P24090	ENSRNOG00000038370	ENSG00000145192
RATKERK5B	NCBI M93638	-	ENSG00000186081
RBM13	Uniprot Q9BXY0	ENSRNOG00000010783	ENSG00000198042
Renbp	Uniprot P51607	ENSRNOG00000037267	ENSG00000102032
Scd2	Uniprot Q6P7B9	ENSRNOG00000013279	ENSG00000099194
Sdc3	Uniprot P33671	ENSRNOG00000011927	ENSG00000162512
SEC14L1	Uniprot Q92503	ENSRNOG00000002722	ENSG00000129657
SEC24A	Uniprot O95486	ENSRNOG00000004563	ENSG00000113615
SEC24B	Uniprot O95487	ENSRNOG00000023373	ENSG00000138802
SERGEF	Uniprot Q9UGK8	ENSRNOG00000011488	ENSG00000129158
Shank2	Uniprot Q9QX74	ENSRNOG00000029931	ENSG00000162105
SHFM1	Uniprot P60896	ENSRNOG00000010420	ENSG00000127922
Slc10a2	Uniprot Q62633	ENSRNOG00000037753	ENSG00000125255
Slc18a3	Uniprot Q62666	ENSRNOG00000025008	ENSG00000187714

Slc1a2	Uniprot P31596	ENSRNOG00000005479	ENSG00000110436
Slc2a13	Uniprot Q921A2	ENSRNOG00000015741	ENSG00000151229
Slc30a1	Uniprot Q62720	ENSRNOG00000004749	ENSG00000170385
SOD1	Uniprot P07632	ENSRNOG00000002115	ENSG00000142168
SPARC	EMBL X04017	ENSRNOG00000012840	ENSG00000113140
Ssg1	EMBL AA870126	ENSRNOG00000002052	ENSG00000091986
SSR3	Uniprot Q9UNL2	ENSRNOG00000011148	ENSG00000114850
Sstr3	Uniprot P30936	ENSRNOG00000007332	ENSG00000183473
STMN1	Uniprot P16949	ENSRNOG00000016810	ENSG00000117632
Strn	Uniprot P70483	ENSRNOG00000004806	ENSG00000115808
Sult1b1	Uniprot P52847	ENSRNOG00000001967	ENSG00000173597
Tcfap2a	EMBL X57012	ENSRNOG00000015522	ENSG00000137203
Thbs2	EMBL L07803	ENSRNOG00000010529	ENSG00000186340
TIS11	EMBL X14678	ENSRNOG00000019673	ENSG00000128016
Tmod	EMBL S76831	ENSRNOG00000009761	ENSG00000136842
Tspan15	EMBL BC003872	-	ENSG00000099282
TUFM	Uniprot P49411	ENSRNOG00000018604	ENSG00000178952
Ucp2	EMBL AF111998	ENSRNOG00000017854	ENSG00000175567
Unkl	-	ENSRNOG00000017598	ENSG00000059145
USP10	Uniprot Q14694	ENSRNOG00000016509	ENSG00000103194
Utp14a	Uniprot Q5M811	ENSRNOG00000005012	ENSG00000156697
Vac14	-	ENSRNOG00000017219	ENSG00000103043
Vat1	EMBL X95562	ENSRNOG00000020684	ENSG00000108828
Vegf	Uniprot P16612	ENSRNOG00000019598	ENSG00000112715
Vps24	Uniprot Q8CGS4	ENSRNOG00000007356	ENSG00000115561
VPS28	Uniprot Q9UK41	ENSRNOG00000014633	ENSG00000160948
VPS29	Uniprot Q9UBQ0	ENSRNOG00000001274	ENSG00000111237
VPS41	Uniprot P49754	ENSRNOG00000012940	ENSG00000006715
Wipi2	Uniprot Q6AY57	ENSRNOG00000001114	ENSG00000157954
Xdh	EMBL X62932	ENSRNOG00000007081	ENSG00000158125
YEATS4	Uniprot O95619	ENSRNOG00000005689	ENSG00000127337

The sequences encoding shRNAs used in the screen were cloned into the pSuper vector and are listed in Supplementary Table 1 (Online Resource 2).

shRNA library screen

In screening experiments, cortical neurons were transfected on Day 6 *in vitro* (DIV6) with TSC2sh together with pools of pSuper plasmids that encoded shRNAs targeting a given gene and β-actin-GFP in the following proportions: 0.35/0.15/0.15/0.15/0.22 (TSC2sh/shRNA#1/#2/#3/GFP) or 0.35/0.22/0.22/0.22 (TSC2sh/shRNA#1/#2/GFP), when only two shRNAs were used to silence a given mRNA. Each culture plate contained 3 control variants: (i) transfected with pSuper/β-actin-GFP (0.8/0.22), (ii) transfected with TSC2sh/pSuper/β-actin-GFP (0.35/0.45/0.22), and (iii) transfected with TSC2sh/pSuper/β-actin-GFP and treated with 20 nM rapamycin.

Western blot

Cells were lysed in 20 mM Tris pH 7.5, 150 mM NaCl, 2 mM EDTA, 0.5% Igepal, 0.5% Triton-X, 2 mM $MgCl_2$, 10% glycerol, supplemented with protease and phosphatase inhibitors (Roche, Basel, Switzerland). Protein concentration was measured with the Pierce BCA Protein Assay Kit (Thermo Scientific, Waltham, MA). Samples were boiled after adding 4× Laemmli buffer and equal amounts of proteins were loaded onto the gel. Proteins from cell extracts were separated according to size by SDS-PAGE and transferred to a nitrocellulose membrane using a transfer apparatus according to the manufacturer's protocols (Bio-Rad, Hercules, CA). When horseradish peroxidase-conjugated secondary antibodies were used, after incubation with 5% nonfat milk in TBS-T (10 mM Tris, pH 8.0, 150 mM NaCl, 0.1% Tween 20) for 60 min, the membrane was washed 3 times with TBS-T and incubated with the appropriate primary antibodies diluted in TBS-T with 5% nonfat milk or bovine serum albumin (BSA) at 4°C overnight. Then, the membranes were washed 3 times with TBS-T and incubated with horseradish peroxidase-conjugated anti-mouse or anti-rabbit antibodies in

TBS-T with 5% nonfat milk for 1 h. The blots were washed with TBS-T and developed with 1.25 mM luminol, 200 μM coumaric acid, and 0.01% H_2O_2 in 100 mM Tris pH 8.5. In case of signal detection with Infrared Odyssey Imaging System (LI-COR Biosciences), membrane was first incubated for 60 min with 5% nonfat milk in TBS (10 mM Tris, pH 8.0, 150 mM NaCl), washed 3 times with TBS and incubated with the appropriate primary antibodies diluted in TBS-T with 5% nonfat milk or BSA at 4°C overnight. Then, the membranes were washed 3 times with TBS-T and incubated with anti-mouse IRDye680 and anti-rabbit IRDye800 antibodies (LI-COR Biosciences) in TBS-T with 5% nonfat milk for 1h. The blots were washed with TBS-T and water and let to dry completely before fluorescence signal acquisition with use of Infrared Odyssey Imaging System.

In vivo electroporation in neonates

Neonates (P0; Wistar) were anesthetized by hypothermia (5 min). Subsequently, animals were placed in a stereotaxic apparatus under a Hamilton syringe connected to a pulled out glass capillary (beveled to a <50 μm diameter, GC100-15, Clark, UK). The syringe was positioned at the level of the skull, then lowered 2.5 mm into the lumen of the right lateral ventricle and the plasmid solution (3 μg/μl diluted in PBS containing 0.1% Fast Green, Sigma, St Louis, MO) was injected. Plasmid solution contained mix of plasmids encoding shRNA with pCx EGFP-N1. Successfully injected animals were subjected to 5 electrical pulses (50 ms, separated by 950 ms intervals) using the CUY21 device (Nepagene, Chiba, Japan) and 10 mm tweezer electrodes (CUY650P10, Nepagene). Electroporated animals were warmed on a heating pad for several minutes before being returned to the mother.

Rat tissue preparation and staining

Animals were transcardially perfused with phosphate buffered saline (PBS) and then with 4% PFA in phosphate buffer (pH = 7). The brains were isolated and postfixed for 2 h in PFA. Postfixed brains were cryopreserved in 30% sucrose, frozen, and 100 μm sections cut on a

cryostat. Immunofluorescence was performed on free-floating sections. The brain sections were incubated at 4°C overnight with anti-GFP antibody in PBS, 0.1% Triton-X, and 1% donkey serum. Then, the sections were rinsed 3 times with PBS and incubated for 1 h at room temperature with secondary antibody conjugated to fluorochrome to visualize immunostaining. Lastly, the sections were rinsed, mounted on slides, and coverslipped prior to imaging.

Human tissue preparation and staining

Formalin fixed, paraffin-embedded tissue (one representative paraffin block per case containing the complete lesion or the largest part of the lesion resected at surgery) was sectioned at 6 µm and mounted on pre-coated glass slides (Star Frost, Waldemar Knittel GmbH, Barunschweig, Germany). Sections of all specimens were processed for hematoxylin-eosin (HE), luxol fast blue (LFB), and Nissl stains, as well as for immunohistochemical staining for a number of neuronal and glial markers (not shown). Immunohistochemistry on human samples was carried out as previously described (Aronica *et al.*, 2003). Single-label immunohistochemistry was performed using the Powervision kit (Immunologic, Duiven, The Netherlands) and 3,3-diaminobenzidine as chromogen. Sections were counterstained with hematoxylin.

Image acquisition

Olympus Cell^R station equipped with 20× objective was used to obtain neuron images in screening experiments and for live imaging of SEGA-derived cells. Confocal images of immunofluorescently stained cortical neurons and rat brain sections were obtained with a Zeiss LSM710NLO microscope, equipped with 20× objective, at 1024 × 1024 pixel resolution. Each image consisted of a series of *z*-stack images. For images of cortical neurons cultured *in vitro*, the resultant stack was flattened into a single image using maximum projection. In the case of images of rat brain slices, 3D reconstruction was performed. The

confocal settings were constant for all of the scans when fluorescence intensity was compared.

Sample sizes for experimental groups

The exact numbers of analyzed cells/animals for each experimental condition are provided in the table below.

Sample sizes for experimental groups

	**Figure 1a, P-S6 intensity**						
experimental variant	TSC2sh:	-	#1	#2	-	#1	#2
	rapamycin:	-	-	-	+	+	+
number of values		55	58	48	56	56	57

	**Figure 1a, cell soma area**						
experimental variant	TSC2sh:	-	#1	#2	-	#1	#2
	rapamycin:	-	-	-	+	+	+
number of values		58	59	68	63	62	55

	**Figures 1d and 1e**												
experimental variant	TSC2sh:	-	-	-	-	#1	#1	#1	#1	#2	#2	#2	#2
	GCLCsh:	-	#1	#3	mix	-	#1	#3	mix	-	#1	#3	mix
number of values		57	53	64	57	54	56	61	57	58	65	59	58

	**Figure 2**			
experimental variant	TSC2sh:	-	+	+
	GCLCsh:	-	-	+
number of values		161	159	245
number of animals		6	5	7

	**Figure 3a**						
experimental variant	TSC2sh:	-	#1	#2	-	#1	#2
	L-BSO:	-	-	-	+	+	+
number of values		133	119	125	85	85	130

	**Figure 5a, SEGA#1**					
experimental variant	control	rapamycin	rapamycin UO126	L-BSO 20 µm	L-BSO 100 µm	rapamycin L-BSO 20 µm
number of values	61	69	64	62	48	36

	**Figure 5a, SEGA#2**					
experimental variant	control	rapamycin	rapamycin UO126	L-BSO 20 µm	L-BSO 100 µm	rapamycin L-BSO 20 µm
number of values	52	78	92	59	53	51

	**Figure 6b, SEGA#1**

experimental variant	control	rapamycin	rapamycin UO126	L-BSO 20 µm	L-BSO 100 µm	rapamycin L-BSO 20 µm
number of values	88	105	104	106	106	100
	**Figure 6b, SEGA#2**					
experimental variant	control	rapamycin	rapamycin UO126	L-BSO 20 µm	L-BSO 100 µm	rapamycin L-BSO 20 µm
number of values	66	78	66	71	70	99
