# Supplementary material for: Tuberous sclerosis complex neuropathology requires glutamate-cysteine ligase
Source: Acta Neuropathol Commun. 2015 Jul 30;3:48. doi: 10.1186/s40478-015-0225-z (PMC4518593; doi:10.1186/s40478-015-0225-z)
Supplement: Supplementary file 3 — Supplementary Figures and Table S2. (PDF 504 kb) [file 40478_2015_225_MOESM3_ESM.pdf]

Online Resource 3

Title: Tuberous sclerosis complex neuropathology requires glutamate-cysteine ligase
Malik *et al.*

Supplementary Figures and Tables

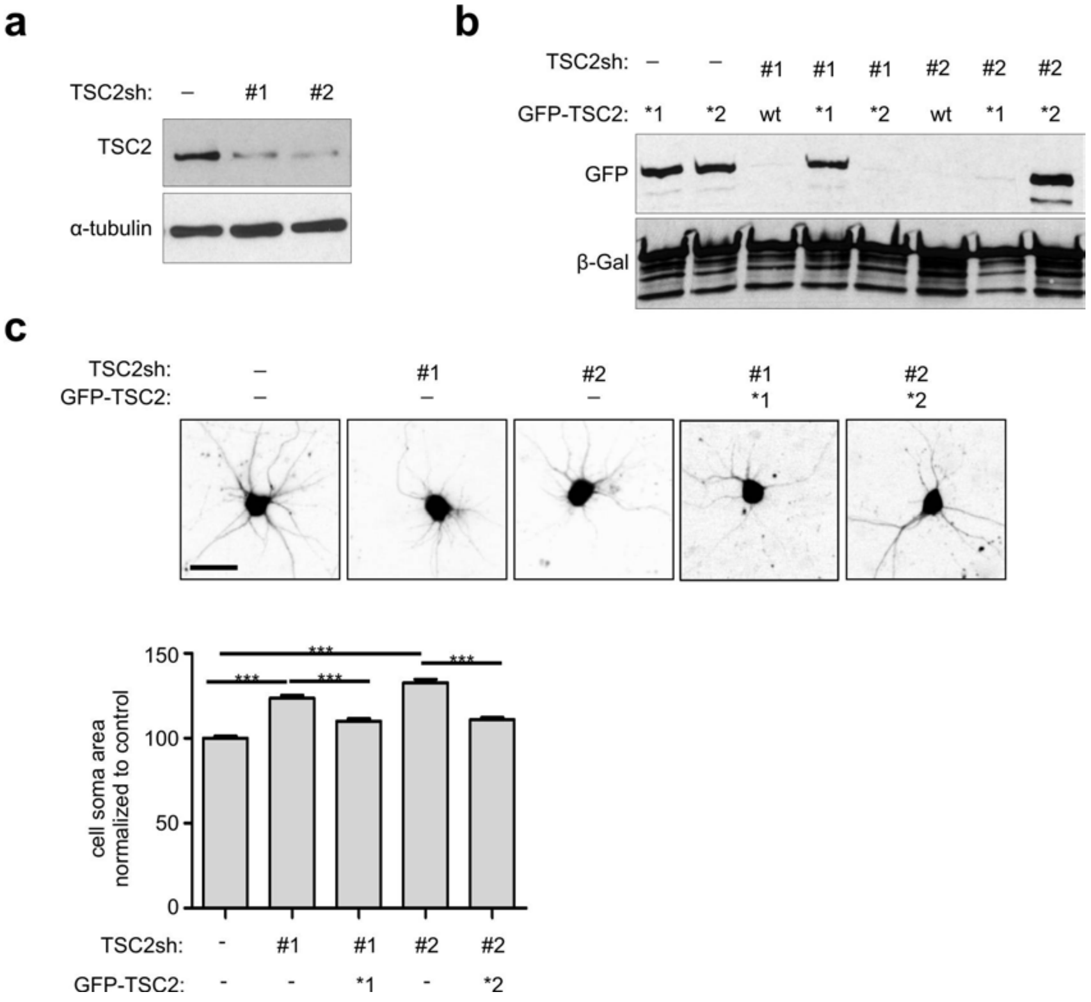

Supplementary Figure 1. Evaluation of shRNA tools.

(**a**) Western blot analysis of TSC2 protein level in cortical neurons nucleofected with empty pSuper vector or TSC2sh#1 or #2 2 days after nucleofection. α-tubulin is shown as a loading control.

(**b**) Western blot analysis of GFP-tagged TSC2 levels in COS-7 cell lysate. Cells were transfected with empty pSuper vector, TSC2sh#1, or TSC2sh#2 together with shRNA-resistant mutants of GFP-tagged TSC2. GFP-TSC2*1 and GFP-TSC2*2 are resistant to TSC2sh#1 and #2, respectively. Plasmid encoding β-galactosidase (β-Gal) was co-transfected to indicate transfection levels.

(**c**) Representative images and quantification of cell soma area of cortical neurons transfected with empty pSuper vector, TSC2sh#1, or TSC2sh#2, together with a plasmid encoding GFP, GFP-TSC2*1, or GFP-TSC*2. Scale bar: 50 µm. The plot represents mean ± SEM. ***p < 0.001 in Kruskal-Wallis with Dunn's post-hoc test. Sample sizes (number of cells) for experimental groups are following: pSuper (114), TSC2sh#1 (96), TSC2sh#1/ GFP-TSC2*1 (105), TSCsh#2 (97), TSCsh#2/ GFP-TSC*2 (119).

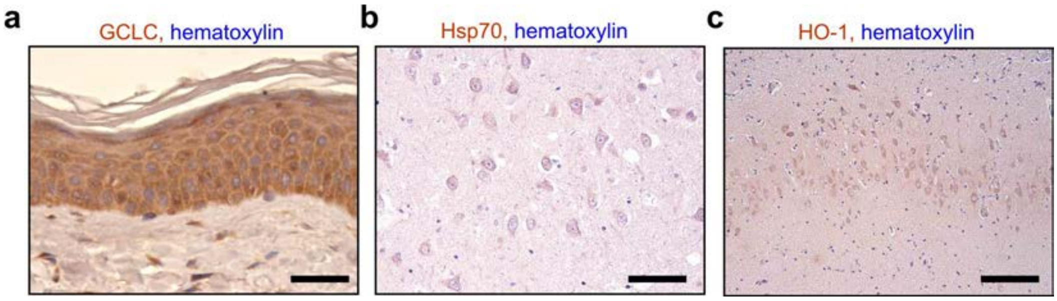

Supplementary Figure 2. Human tissue staining: positive controls.

(**a**) GCLC immunoreactivity in squamous epithelial cells. Scale bar: 40 µm

(**b**) Hsp70 immunoreactivity in hippocampal neurons (CA1) of a patient with traumatic brain injury. Scale bar: 80 µm.

(**c**) HO-1 immunoreactivity in hippocampal neurons (CA1) of a patient with Alzheimer's disease. Scale bar: 160 µm.

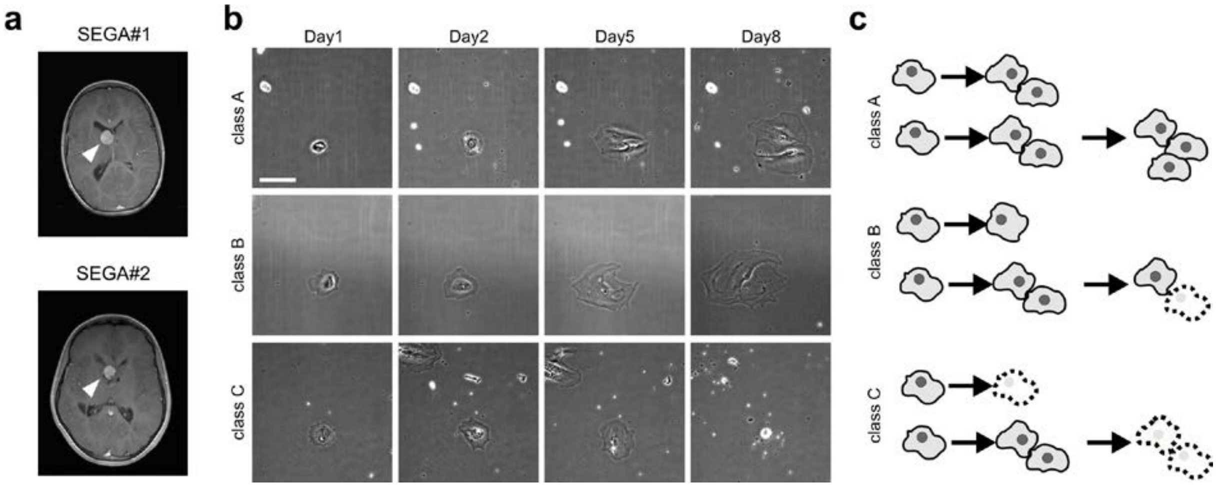

Supplementary Figure 3. SEGA-derived cells live imaging.

(**a**) Patients' brain MRI, arrowheads point to SEGAs that were surgically resected and used to obtain SEGA-derived cell cultures.

(**b**) Exemplary images of SEGA-derived cells in given days of live imaging that were later assigned class A, B, or C. Scale bar: 100 µm

(**c**) Schematic representation of cell fates that were assigned given cell classes.

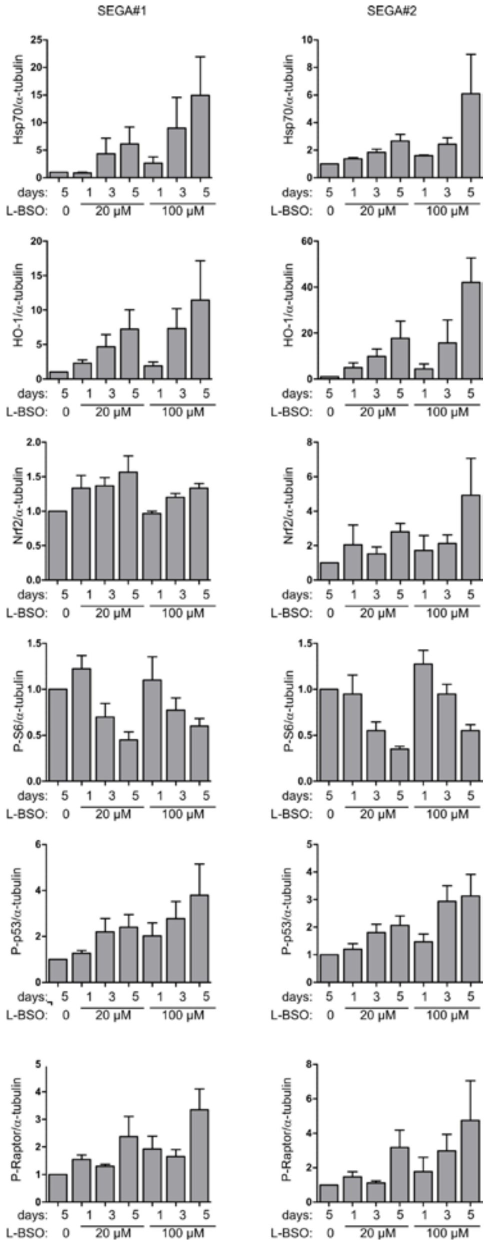

Supplementary Figure 4. Quantitative analysis of effects of GCLC inhibition in SEGA-derived cells on Hsp-70, HO-1, Nrf2, P-S6 (Ser 235/236), P-p53 (Ser 15) and P-Raptor (Ser 792). Western blot membranes shown in Fig 5 were scanned using an Odyssey CLx infrared imaging system (LI-COR Biosciences) with two-color fluorescence detection at 700 and 800 nm, quantified using LICOR Western Blot Analysis Software - Image Studio Lite Ver 5.0 on raw unprocessed data files and normalized to α-tubulin.

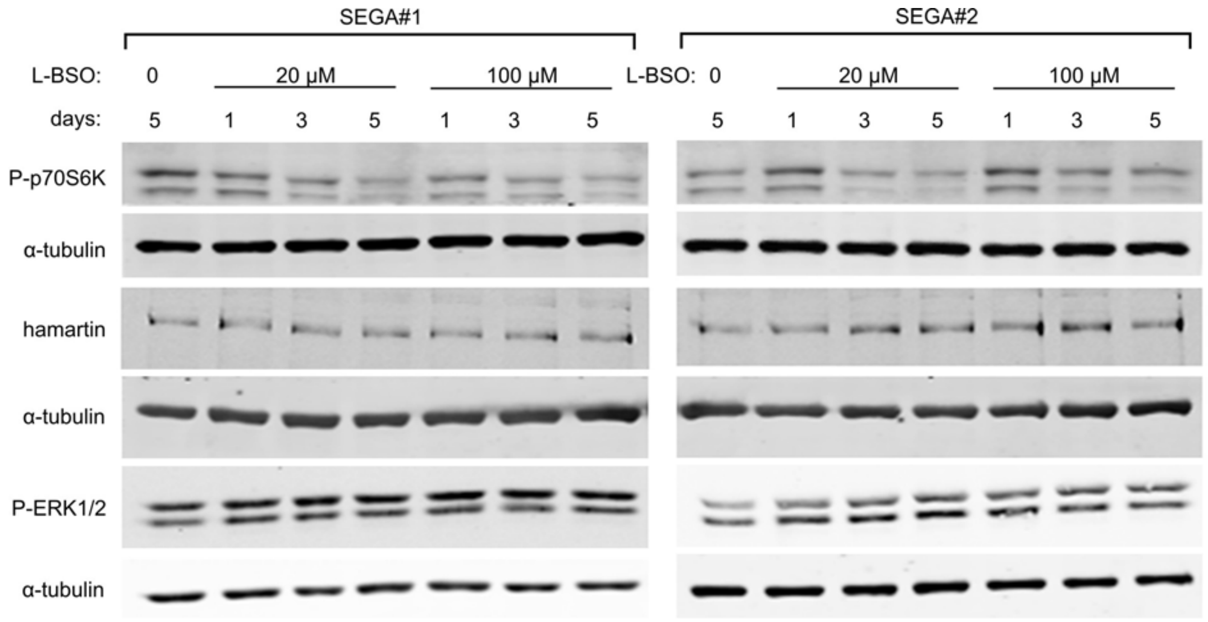

Supplementary Figure 5. GCLC inhibition decreases levels of phosphorylated p70S6K (Thr 389) but not phosphorylated ERK1/2 (Thr201/Tyr204) or hamartin in SEGA-derived cells. Western blot analysis of p70S6K (Thr 389) phosphorylated ERK1/2 (Thr201/Tyr204) and hamartin in SEGA-derived cells lysed after a 1-, 3- or 5-day treatment with 20 or 100 μM L-BSO. α-tubulin is shown as a loading control.

Supplementary Table 1. Sequences of shRNAs used for the screen (separate file; Online Resource 2)

Supplementary Table 2. Positive hits from screening experiments.

			TSC2sh#1			TSC2sh#2		
shRNA pool	protein	Gene Ensembl ID or *protein Uniprot ID*	Neuron soma area [% pSuper]	SEM	St	Neuron soma area [% pSuper]	SEM	St
13485	ALG5	ENSRNOG00000013485	82.76	3.89	***	101.9	1.931	***
anxa6	ANXA6	ENSRNOG00000010668	84.09	1.59	***	112.9	2.371	***
38370	RATBSP	ENSRNOG00000038370	86.52	1.91	***	111.2	2.125	***
11352	Furin	ENSRNOG00000011352	87.09	2.26	**	108.7	2.152	***
56931	Vps8	ENSRNOG00000001764	87.94	1.30	***	89.93	1.604	***
09988	Ap2b1	ENSRNOG00000009988	88.85	2.40	***	104.7	1.578	***
09545	Polr1a	ENSRNOG00000009545	89.84	3.62	***	98.21	1.856	***
25430	DDX18	ENSRNOG00000025430	90.74	2.38	***	93.13	1.895	***

23856	Agxt	ENSRNOG00000023856	91.00	1.55	***	100.5	1.988	***
17598	Unkl	ENSRNOG00000017598	91.32	2.94	***	105.8	1.878	***
06302	Gclc	ENSRNOG00000006302	92.03	3.57	**	107.6	2.253	*
19601	Mapk3	ENSRNOG00000019601	92.81	3.96	**	95.94	1.798	***
10326	Arfgap1	ENSRNOG00000010326	93.47	2.13	***	99.23	1.969	***
19598	Vegf	ENSRNOG00000019598	94.21	3.22	**	101	1.774	**
12278	Fgf10	ENSRNOG00000012278	94.96	1.75	**	110.1	2.204	***
25008	Slc18a3	ENSRNOG00000025008	95.64	1.44	**	103.7	2.436	***
Fath2	FATh2	ENSRNOG00000012575	96.32	2.58	**	106.5	2.092	**
30445	Ormdl3	ENSRNOG00000030445	96.78	2.77	***	104.4	1.554	**
tcfap2a	Tcfap2a	ENSRNOG00000015522	96.79	2.45	**	108.7	1.694	***
Assr	Ass	ENSRNOG00000008837	97.18	2.14	*	103.8	2.223	***
98841	KTI12	Q5I0L7	96.81	1.64	***	98.38	1.633	***
19959	Kcnc3	ENSRNOG00000019959	100.7	2.191	***	103.5	1.512	***
15145	Ppp6c	ENSRNOG00000015145	102	1.425	**	109.4	2.288	***

St. - statistical significance in Kruskall-Wallis test with post-hoc Dunn's test compared to TSC2sh; ***p<0.001, **p<0.01, *p<0.05.
